# 400. Exploring the Evolving Landscape of Dermatophytosis: Insights from a Case Series on Trichophyton indotineae and Related Genotypes Emerging in the UAE

**DOI:** 10.1093/ofid/ofaf695.138

**Published:** 2026-01-11

**Authors:** Fatima Al Dhaheri, Hari Vanam, Dana Aljneibi, Akela Ghazawi, Febin Anes, Fouzia Jabeen, Mushtaq Khan, Ahmed R Alsuwaidi, Jens Thomsen, Mohammad AlBataineh, Stefan Weber, Connie Gibas, Nathan P Wiederhold

**Affiliations:** United Arab Emirates University, Abu Dhabi, Abu Dhabi, United Arab Emirates; UAEU, Al Ain, Abu Dhabi, United Arab Emirates; United Arab Emirates University, Abu Dhabi, Abu Dhabi, United Arab Emirates; United Arab Emirates University, Abu Dhabi, Abu Dhabi, United Arab Emirates; UAEU, Al Ain, Abu Dhabi, United Arab Emirates; Purelab, Abu Dhabi, Abu Dhabi, United Arab Emirates; United Arab Emirates University, Abu Dhabi, Abu Dhabi, United Arab Emirates; United Arab Emirates University, Abu Dhabi, Abu Dhabi, United Arab Emirates; Medics Labor AG, Khalifa University (adjunct), Bern, Bern, Switzerland; Yarmouk University/George Washington (adjunct) University/Khalifa University (adjunct), Irbid, Irbid, Jordan; Purelab, Abu Dhabi, Abu Dhabi, United Arab Emirates; UT Health San Antonio, San Antonio, Texas; University of Texas Health San Antonio, San Antonio, TX

## Abstract

**Background:**

Dermatophytosis is one of the most common superficial fungal infections globally, increasingly complicated by the emergence of antifungal-resistant strains such as *Trichophyton indotineae*. A member of the *T. mentagrophytes* complex, *T. indotineae* is particularly notable for terbinafine resistance, a frontline oral antifungal. Despite its global spread, limited data exist on the genetic and resistance profiles of *T. indotineae* and related genotypes in the UAE. This study aimed to characterize the molecular and epidemiological landscape of dermatophytosis in the region.

**Figure 1.**
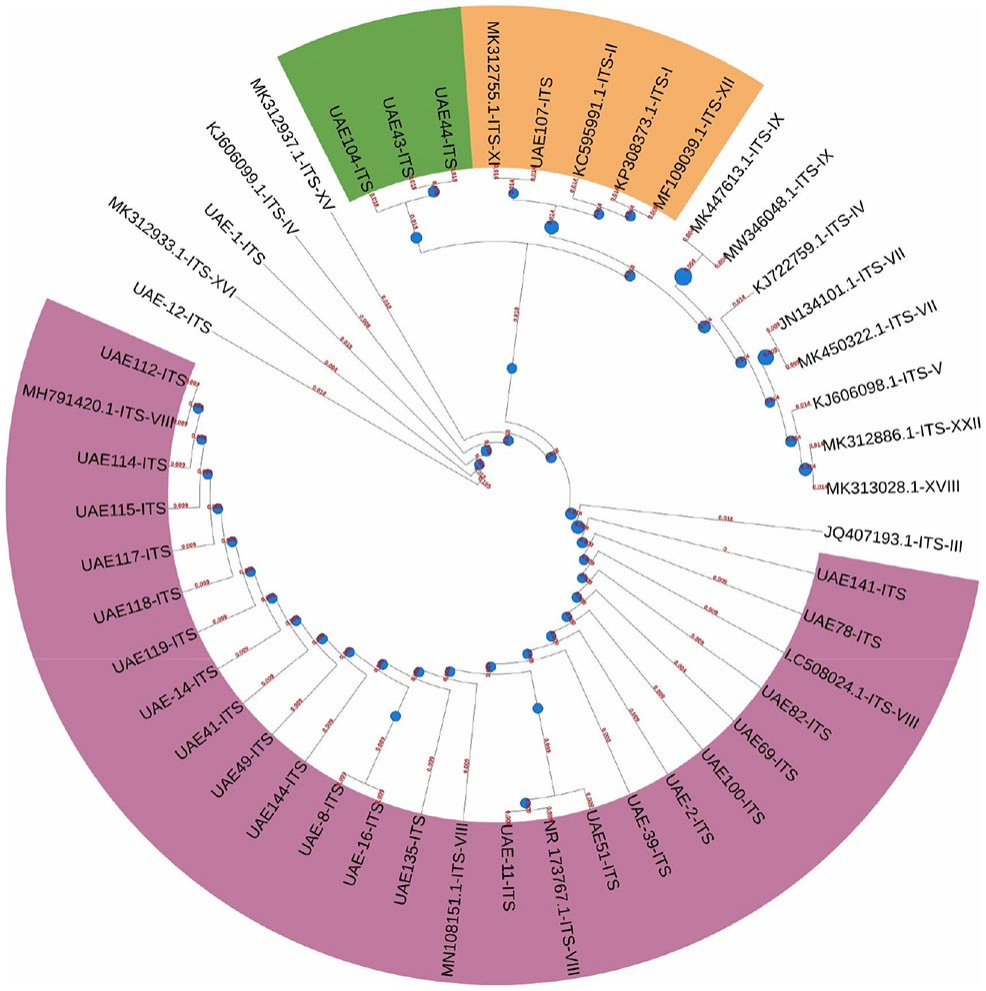
Maximum likelihood phylogenetic tree based on ITS sequences illustrating the relationships among UAE isolates and reference sequences. The tree is color-coded by clade: green, orange, and purple, indicating distinct ITS lineages or genotypes. Red numbers represent branch lengths, corresponding to the evolutionary distance between sequences. Blue dots mark nodes with high bootstrap support, indicating confidence in the phylogenetic grouping.

**Figure 2.**
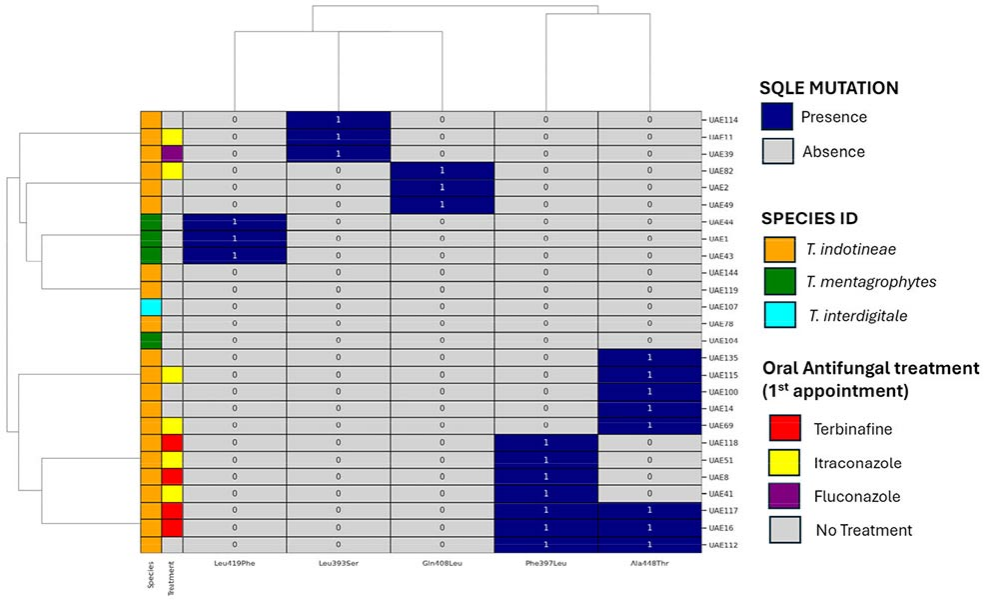
Clustered heatmap showing amino acid substitutions in the SQLE gene among UAE clinical isolates. Each row represents a UAE isolate, and each column corresponds to a specific mutation (e.g., Leu393Ser, Phe397Leu, Gln408Leu, Leu419Phe, Ala448Thr). Blue cells indicate the presence of a mutation (1), and grey cells represent absence (0).

**Methods:**

From September 2024 to February 2025, 26 clinical dermatophyte isolates from tinea cases were collected across the Abu Dhabi Emirate. Clinical metadata were reviewed. Isolates underwent macroscopic/microscopic examination, urease testing, internal transcribed spacer (ITS) genotyping, and sequencing of the squalene epoxidase (SQLE) gene to identify terbinafine resistance mutations. Phylogenetic analysis was performed using reference ITS genotypes.

**Results:**

Of the 26 isolates, 21 (80%) were identified as *T. indotineae* (ITS genotype VIII), four as *T. mentagrophytes*, and one as *T. interdigitale*. While morphologically diverse, micromorphology was similar across isolates. SQLE mutations were found in 21 isolates (80.7%), with the Phe397Leu/Ala448Thr double mutation most common. Risk factors included topical steroid misuse, animal contact, and inadequate systemic antifungal therapy. Phylogenetic analysis showed clustering into three groups, indicating local transmission and clonal expansion. Sequences were submitted to GenBank.

**Conclusion:**

A high prevalence of terbinafine-resistant *T. indotineae* was observed among dermatophytosis cases in the UAE. The presence of multidrug-resistant genotypes and local transmission highlights the urgent need for accurate fungal identification, routine antifungal susceptibility testing, public education, and antifungal stewardship to curb the spread of recalcitrant dermatophytosis.

**Disclosures:**

All Authors: No reported disclosures

